# Supplementary material for: Modeling-Based Decision Support System for Radical Prostatectomy Versus External Beam Radiotherapy for Prostate Cancer Incorporating an In Silico Clinical Trial and a Cost–Utility Study
Source: Cancers (Basel). 2021 May 29;13(11):2687. doi: 10.3390/cancers13112687 (PMC8198879; doi:10.3390/cancers13112687)
Supplement: Supplementary file 1 [file cancers-13-02687-s001.zip › Supplementary Document I.pdf]

Supplementary Document I

Within the Markov model used for the decision support system (DSS), the health state "disease free" consists a number of sub-heath states, namely no toxicity, or any combination of three different toxicities: rectal bleeding, urinary incontinence and erectile dysfunction. From the models we have available the probability that a patient has one of three different toxicities after a certain amount of time, however we need to transfer this into transition probabilities within our model. To explain how these were calculated please refer to the diagram in Figure A 1 below.

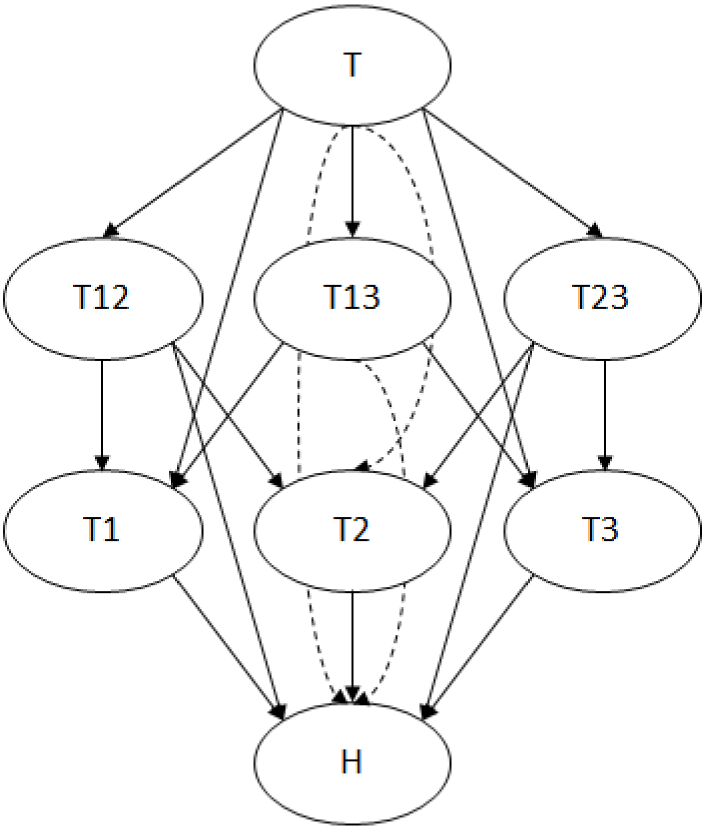

Figure A 1 - The transition probability diagram of the "Healthy" life state. T = all three toxicities; H = no toxicities; T1 = erectile dysfunction; T2 = rectal bleeding; T3 = urinary incontinence; T12 = erectile dysfunction and rectal bleeding; T13 = erectile dysfunction and urinary incontinence; T23 = rectal bleeding and urinary incontinence

Here we see that when patients have a toxicity, they have a probability to recover from toxicity, but they will never get a toxicity if they don't have it initially. We now need to calculate the separate transition probabilities using the probability of a patient recovering from a certain toxicity. First, we convert the probability calculated from the nomograms to the probability per cycle.

P1 = probability of recovering from Erectile dysfunction per cycle

P2 = probability of recovering from Rectal bleeding per cycle

P3 = probability of recovering from Urinary incontinence per cycle

First we will calculate the probability of going from all toxicities to any other health state, as shown in Figure A 2 below.

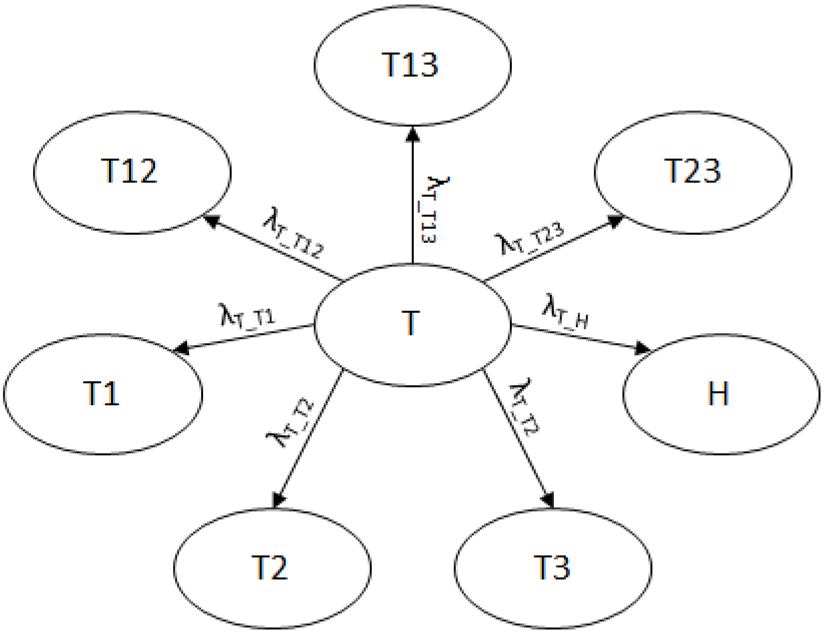

Figure A 2 - A diagram showing the transition from all toxicities health state to all other health states

The probability of recovering from all three toxicities in one cycle can be obtained using the following relation:

$$\lambda_{T\_H} = P1 * P2 * P3$$

The probability of recovering from two different toxicities can be calculated by multiplying the probability of recovering from either toxicity, subtracted by the probability of recovering from all three toxicities.

$$\lambda_{T\_T1} = P2 * P3 - \lambda_{T\_H}$$

$$\lambda_{T\_T2} = P1 * P3 - \lambda_{T\_H}$$

$$\lambda_{T\_T3} = P1 * P2 - \lambda_{T\_H}$$

The probability of recovering from only one toxicity is equal to the total probability of recovering of that toxicity subtracted by the probability of recovering from that toxicity in combination with any other toxicity.

$$\lambda_{T\_T23} = P1 - \lambda_{T\_T2} - \lambda_{T\_T3} - \lambda_{T\_H}$$

$$\lambda_{T\_T13} = P2 - \lambda_{T\_T1} - \lambda_{T\_T3} - \lambda_{T\_H}$$

$$\lambda_{T\_T12} = P3 - \lambda_{T\_T1} - \lambda_{T\_T2} - \lambda_{T\_H}$$

Next we want to calculate the state transition probabilities from a combination of two toxicities to healthy or to a single toxicity, as shown in Figure A 3 below.

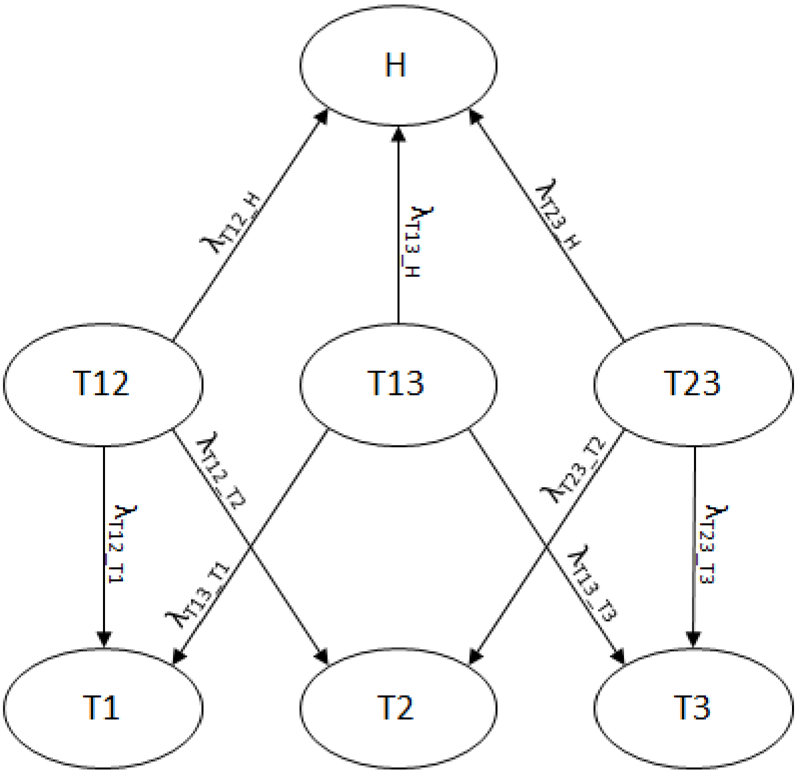

Figure A 3 - A diagram showing the state transitions from any combination between two toxicities to the no toxicity health state and a single toxicity heath state

The transition probability of any combination of two toxicities two healthy is multiplication of the total probability of recovering of either toxicity:

$$\lambda_{T12\_H} = P1 * P2$$

$$\lambda_{T13\_H} = P1 * P3$$

$$\lambda_{T23\_H} = P2 * P3$$

The probability of recovering from one of the two toxicities is the total probability of recovering from that toxicity subtracted by the probability of recovering from both toxicities.

$\lambda_{T12\_T1} = P1 - \lambda_{T12\_H}$

$\lambda_{T12\_T2} = P1 - \lambda_{T12\_H}$

$\lambda_{T13\_T1} = P1 - \lambda_{T13\_H}$

$\lambda_{T13\_T3} = P3 - \lambda_{T13\_H}$

$\lambda_{T23\_T2} = P2 - \lambda_{T23\_H}$

$\lambda_{T23\_T3} = P3 - \lambda_{T23\_H}$

Finally, the most straightforward transition probability is the probability of recovering from a single toxicity, which is equal to the total probability of recovering from that toxicity, as shown in Figure A 4.

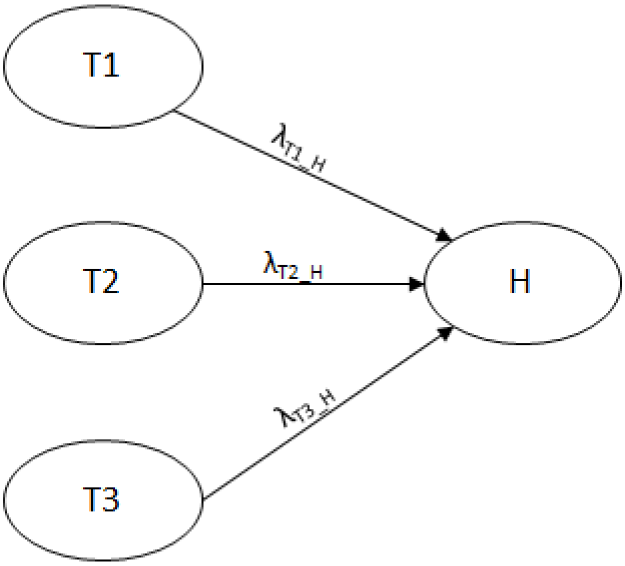

Figure A 4 - The diagram showing the transition from a single toxicity health states to the no toxicity heath state

Therefore:

$\lambda_{T1\_H} = P1$

$\lambda_{T2\_H} = P2$

$\lambda_{T3\_H} = P3$
