# Supplementary material for: Modeling-Based Decision Support System for Radical Prostatectomy Versus External Beam Radiotherapy for Prostate Cancer Incorporating an In Silico Clinical Trial and a Cost–Utility Study
Source: Cancers (Basel). 2021 May 29;13(11):2687. doi: 10.3390/cancers13112687 (PMC8198879; doi:10.3390/cancers13112687)
Supplement: Supplementary file 1 [file cancers-13-02687-s001.zip › Supplementary Document II.pdf]

Supplementary Document II

An overview of the utility values, obtained from Steward et al. are shown in Table A 1.

Table A 1- Utility values used in the Markov model

State	Expected value	SD
Post-treatment, no adverse effects	Baseline	-
ED	0.89	0.16
UI	0.83	0.21
RB	0.71	0.26
UI+BT	0.70	0.24
ED+BT	0.57	0.26
ED+UI	0.79	0.23
UI+ED+BT	0.45	0.31
Recurrence	0.81	0.18
Metastatic disease	0.25	0.11

ED: erectile dysfunction; UI: Urinary incontinence; RB: rectal bleeding; SD: standard deviation

An overview of the costs are shown in Table A 2

Table A 2 - Costs used in the Markov model

Description	Mean costs (€)	SD (€)
Open prostatectomy	9780	978
Radiotherapy	8275	827.5
	**Mean costs per year (€)**	**SD (€)**
Follow-up (year 1-2)	511	30
Follow-up (year >2)	255	21
Add. costs erectile dysfunction	60	9
Add. costs urinary incontinence	303	21
Add. costs rectal bleeding	95	10
Recurrence	2535	212
Metastatic disease	2288	252
Castrate resistant metastasis	6493	313

SD = standard deviation

These values were obtained using the care product codes of the relevant treatments. We used the costs published on https://www.opendisdata.nl, and we used a standard deviation of 10% of the total costs of the treatment (Table A 3).

Table A 3- costs of treatment

	Total costs	*Care product code*
Radiotherapy	€ 8275	990061072, 990061014
Open Prostatectomy	€ 9780	20109012

Costs of treatments less than € 850 were extracted using the care product code and the tariff tool developed by CZ (https://www.cz.nl/service-en-contact/zoek-tarieven). For post-treatment costs we assumed 4 consultations and prostate specific antigen (PSA) tests per anno in the first two years, and 2 per anno after that (Table A 4).

Table A 4- post-treatment costs

	Quantity p.a		*Price/unit*	*Total costs*		*Care product code*
Year	1-2	>2		1-2	>2	
Consultation fee	4	2	€ 122 (15)	€ 488 (30)	€ 244 (21)	20109088
PSA test	4	2	€ 5,8	€ 23,2	€ 11,6	72621
Total				**€ 511,2 (30)**	**€ 255,6 (21)**	

Costs of medication was taken from a website, https://www.farmacotherapeutischkompas.nl, that reports the costs of pharmaceutical healthcare. At the suspicion of biochemical failure or metastatic disease, a PSMA scan can be made. If recurrence has taken place, the patient can receive secondary localized treatment, or hormonal therapy. Once the patient progresses to castrate resistant metastatic disease, the patient is treated with chemotherapy (Table A 5).

Table A 5- costs metastatic disease

	Quantity p. a	*Price/unit (SD)*	*Total costs (SD)*	*Care product code*
Consultation fee specialist	4	€ 122 (15)	€ 488 (21)	20109088
LHRH agonist leuprorelin	4	€ 450 (125)	€ 1800 (250)	20109010
Docetaxel	6	€ 721 (120)	€ 4326 (294)	20109050
Prednisolon	6	€ 18	€ 108	
PSMA-scan	0,35	€ 1060 (123)	€ 371 (73)	120501
Zoledronic acid	4	€ 422 (40)	€ 1688 (80)	
Total			**€ 8781 (401)**	

SD: standard deviation; p.a; per anno

For the management of late erectile dysfunction, we assumed that half the patients use medication, 10% of the patients use cavernous injections and 5% use a vacuum pump. We estimated that

medication or injections would be used on average once a month, and that the vacuum pump lasts two years (Table A 6)

Table A 6- costs of the management of erectile dysfunction

	Quantity p. a	*Price/unit*	*Total costs*
Sildenafil	6	€ 6,83 (2)	€ 41 (5)
Cavernous injection	1,2	€ 11,06 (3)	€ 13 (3)
Vacuum pump	0,025	€ 235 (35)	€ 6 (6,5)
**Total**			**€ 60 (9)**

For the management of late urinary incontinence, we estimate that approximately three quarters of patients use physiotherapy, with 8 sessions per year. We estimate that 90% of the patients with urinary incontinence use pads 3 times a day. We assume that 5% requires diapers once a day, and all patients who use diapers or pads require net trousers. It is estimated that 2,5% require drainage bags, of which they will require bed bags and leg bags (Table A 7). For the prices of medical products such as pads, net trousers and leg bags, we collected costs from different websites such as www.mediqcombicare.nl, www.pharmamarket.nl, and www.123drogisterij.nl.

Table A 7- costs of the management of urinary incontinence

	Quantity p. a	*Price/unit*	*Total costs*
Physiotherapy	1	€ 35 (0,6)	€ 35 (0,6)
Balloon catheter	0,05	€ 12 (0,3)	€ 0,6 (0,07)
Pads	394	€ 0,36 (1)	€ 142 (20)
Diapers	18	€ 0,65 (0,08)	€ 11,7 (0,3)
Net trousers	0,95	€ 1,25 (0,1)	€ 1,2 (0,1)
Bed bag, sterile	3	€ 20 (0,7)	€ 60 (1,2)
Leg bag, sterile	1,3	€ 40 (1)	€ 52 (1,1)
**Total**			**€ 302,5 (21)**

Rectal bleeding is mostly treated with low cost treatments such as medication, but a low percentage has severe rectal bleeding and needs to be treated with infrared coagulation or a coloscopy (Table A 8)

Table A 8- costs of the management of rectal bleeding

	Quantity p. a	*Price/unit*	*Total costs*	*Care product code*
Salofalk/ Arestal	36,5	€ 1,13 (1)	€ 41 (6)	
Sclerosis, Rubber band ligation, infrared coagulation	0,05	€ 167 (3,8)	€ 8 (0,8)	35135
Intervention-coloscopy	0,05	€ 469 (7)	€ 23,5 (1,6)	34697
Sigmoidoscopy	0,05	€ 445 (34)	€ 22 (8)	34686
**Total**			**€ 94,5 (10)**	
