# Supplementary material for: Modeling-Based Decision Support System for Radical Prostatectomy Versus External Beam Radiotherapy for Prostate Cancer Incorporating an In Silico Clinical Trial and a Cost–Utility Study
Source: Cancers (Basel). 2021 May 29;13(11):2687. doi: 10.3390/cancers13112687 (PMC8198879; doi:10.3390/cancers13112687)
Supplement: Supplementary file 1 [file cancers-13-02687-s001.zip › Supplementary Document III.pdf]

Supplementary Document III – Distribution for the generation of synthetic dataset

Parameter name	*Distribution type*			*Ref*
Age (years)	Normal	μ = 60	σ = 10	[1–5]
PSA (ng/mL)	Beta	α = 4.17	β = 51.5	[1–5]
BMI (kg/m^2)	Normal	μ = 28	σ = 3	[3–5]
T-stage	Step	T1 = 66%	T2 = 34%	[1, 3–5]
neg. biopsy cores (N)	Normal	μ = 4.5	σ = 1.9	[2]
pos. biopsy cores (N)	Half normal	Location = 1	Scale = 2.46	[2]
P. Gleason grade	Beta	4 = 14%	3 = 86%	[1, 3, 4]
S. Gleason grade	Step	4 = 19%	3 = 71%	[2]
ADT given	Step	no = 22%	yes = 78%	[6]
ADT length	Beta	α = 0.125	β = 1.25	[1]
ASA score	Step	I/II = 18%	III/IV = 82%	[1, 3]
Nerve sparing	Step	no = 81%	yes = 19%	[4]
Abdominal surgery	Step	no = 90.3%	yes = 9.7%	[6]
HRQOL (%)	Beta	α = 1.5	β = 6	[5]
Membranous urethra length (mm)	Normal	μ = 12	σ = 3	[4]
Mean Trigone dose (Gy)	Normal	μ = 68	σ = 6	[3]
V75 rectum (%)	Gamma	location = 1	scale = 4	[6]

Ref: reference; PSA: prostate specific antigen; BMI: body mass index; T-stage: Tumor stage; N neg.: number of negative; N pos.: number of positive; P.: Primary; S.: Secondary; ADT: androgen deprivation therapy; ASA: American Society of Anesthesiologists; PN: Pelvic nodes; HRQOL: sexual health-related quality of life; V75: percentage receiving at least 75 Gray; Gy = Gray

1. Warner A, Pickles T, Crook J, et al (2015) Development of ProCaRS Clinical Nomograms for Biochemical Failure-free Survival Following Either Low-Dose Rate Brachytherapy or Conventionally Fractionated External Beam Radiation Therapy for Localized Prostate Cancer. Cureus 7:e276
2. Bjartell A, Bottai M, Persson J, et al (2016) Prediction of clinical progression after radical prostatectomy in a nationwide population-based cohort. Scandinavian Journal of Urology 50:255–259
3. Schaake W, van der Schaaf A, van Dijk LV, et al (2018) Development of a prediction model for late urinary incontinence, hematuria, pain and voiding frequency among irradiated prostate cancer patients. PLoS One 13:e0197757
4. Matsushita K, Kent MT, Vickers AJ, et al (2015) Preoperative predictive model of recovery of urinary continence after radical prostatectomy. BJU Int 116:577–583
5. Alemozaffar M, Regan MM, Cooperberg MR, et al (2011) Prediction of erectile function following treatment for prostate cancer. JAMA 306:1205–1214
6. Valdagni R, Kattan MW, Rancati T, et al (2012) Is it time to tailor the prediction of radio-induced toxicity in prostate cancer patients? Building the first set of nomograms for late rectal syndrome. Int J Radiat Oncol Biol Phys 82:1957–1966
