# Supplementary material for: Modeling-Based Decision Support System for Radical Prostatectomy Versus External Beam Radiotherapy for Prostate Cancer Incorporating an In Silico Clinical Trial and a Cost–Utility Study
Source: Cancers (Basel). 2021 May 29;13(11):2687. doi: 10.3390/cancers13112687 (PMC8198879; doi:10.3390/cancers13112687)
Supplement: Supplementary file 1 [file cancers-13-02687-s001.zip › Supplementary Document IV.pdf]

# Supplementary Document IV – distribution of clinical parameters in synthetic dataset

| *Parameter name* | *EBRT (SD)* | *RP (SD)* | *P* |
|---|---|---|---|
| Age (years) | 63.8 (10.7) | 58.8 (9.1) | <<0.001 |
| PSA (ng/mL) | 8.3 (3.5) | 6.8 (3.5) | <<0.001 |
| BMI (kg/m$^2$) | 27.9 (2.9) | 28.0 (3.0) | 0.81 |
| T-stage 2 (%) | 48.7 | 24.4 | <<0.001 |
| neg. biopsy cores (N) | 3.7 (1.8) | 4.8 (1.8) | <<0.001 |
| pos. biopsy cores (N) | 3.5 (1.7) | 2.7 (1.4) | <<0.001 |
| P. Gleason grade 4 (%) | 27.1 | 3.3 | <<0.001 |
| S. Gleason grade 4 (%) | 14.7 | 20.9 | 0.01 |
| ADT given (%) | 79.7 | 0 | 0.72 |
| ADT length (months) | 9.4 (16.7) | 0 | <<0.001 |
| ASA score >II (%) | 86.2 | 78.9 | 0.003 |
| Nerve sparing (%) | 12.4 | 27.5 | 0.001 |
| Abdominal surgery (%) | 6.4 | 11.6 | 0.005 |
| HRQOL (%) | 82.9 (12.3) | 76.9 (14.6) | <<0.001 |
| Membranous urethra length (mm) | 11.8 (3.0) | 12.1 (2.8) | 0.04 |
| Mean Trigone dose (Gy) | 67.2 (6.0) | 68.4 (5.7) | 0.001 |
| V75 rectum (%) | 3.8 (3.9) | 4.5 (4.6) | 0.008 |

EBRT: external beam radiotherapy; RP: radical prostatectomy; SD: standard deviation; PSA: prostate specific antigen; BMI: body mass index; T-stage: Tumor stage; N neg.: number of negative; N pos.: number of positive; P.: Primary; S.: Secondary; ADT: androgen deprivation therapy; ASA: American Society of Anesthesiologists; PN: Pelvic nodes; HRQOL: sexual health-related quality of life; V75: percentage receiving at least 75 Gray; Gy = Gray
