# Supplementary material for: Modeling-Based Decision Support System for Radical Prostatectomy Versus External Beam Radiotherapy for Prostate Cancer Incorporating an In Silico Clinical Trial and a Cost–Utility Study
Source: Cancers (Basel). 2021 May 29;13(11):2687. doi: 10.3390/cancers13112687 (PMC8198879; doi:10.3390/cancers13112687)
Supplement: Supplementary file 1 [file cancers-13-02687-s001.zip › Supplementary Document V.pdf]

Supplementary Document V – benchmark study including clinical parameters reported

Study	*Treatment [N]*	*Parameters*		*Outcome*	*Value*	*Model*
Hamdy 2016	EBRT [545]	Age (years)	62	5 year BDFS	88.5%	89.0%
		GS 7	21%			
		T2	25%			
		PSA <10	90%			
		PSA ng/ml	4.7			
	RP [553]	Age (years)	62	5 year BDFS	88.1%	87.0%
		GS 7	21%			
		T2	25%			
		PSA <10	90%			
		PSA ng/ml	4.7			
CHHiP	74 Gy [1065]	Age (years)	69	5 year BDFS	88.5%	88.4%
		T2	58%			
		GS 7	64%			
		ADT	83%			
		ADT months	6.2			
	60 Gy [1074]	Age (years)	69	5 year BDFS	91.3%	89.9%
		T2	58%			
		GS 7	63%			
		ADT	85%			
		ADT months	6.2			
	57 Gy [1077]	Age (years)	69	5 year BDFS	86.8%	87.9%
		T2	58%			
		GS 7	65%			
		ADT	84%			
		ADT months	6.2			
Donovan 2016	EBRT [545]	Age (years)	62	6 month UI	5%	3.3%
		GS 7	20%	6 year UI	3.5%	3.3%
		T2	21%	6 month ED	77.8%	85.4%
		PSA (ng/ml)	4.8	6 year ED	72.6%	71.6%
				6 month RB	3.8%	14.7%
				6 year RB	5.6%	5.7%
	RP [553]	Age (years)	62	6 month UI	46%	41.5%
		GS 7	22%	6 year UI	17%	17.0%
		T2	26%	6 month ED	88%	92.7%
		PSA (ng/ml)	4.9	6 year ED	83.5%	83.7%

N: number of patients; EBRT: external beam radiotherapy; RP: radical prostatectomy; BDFS: biological disease free survival; UI: urinary incontinence; ED: erectile dysfunction; RB: rectal bleeding; Gy: gray; GS: gleason score; PSA: prostate specific antigen in ng/ml; ADT: androgen deprivation therapy
