# Supplementary material for: Modeling-Based Decision Support System for Radical Prostatectomy Versus External Beam Radiotherapy for Prostate Cancer Incorporating an In Silico Clinical Trial and a Cost–Utility Study
Source: Cancers (Basel). 2021 May 29;13(11):2687. doi: 10.3390/cancers13112687 (PMC8198879; doi:10.3390/cancers13112687)
Supplement: Supplementary file 1 [file cancers-13-02687-s001.zip › Supplementary Document VI.pdf]

Supplementary Document VI

Univariate one-way sensitivity analyses were performed to examine the impact of individual parameters on the results, of which the fifteen most impactful parameters are graphically represented in a tornado plot (Figure A 1). In the diagram we varied each parameter between the 2.5 and 97.5 percentiles and calculated the resulting iNMB of the DSS compared to the randomized treatment allotment strategy. The most impactful parameter is the utility value of erectile dysfunction, but over the range of these parameters the iNMB of the DSS was still positive, and thus cost-effective when compared to the randomized strategy.

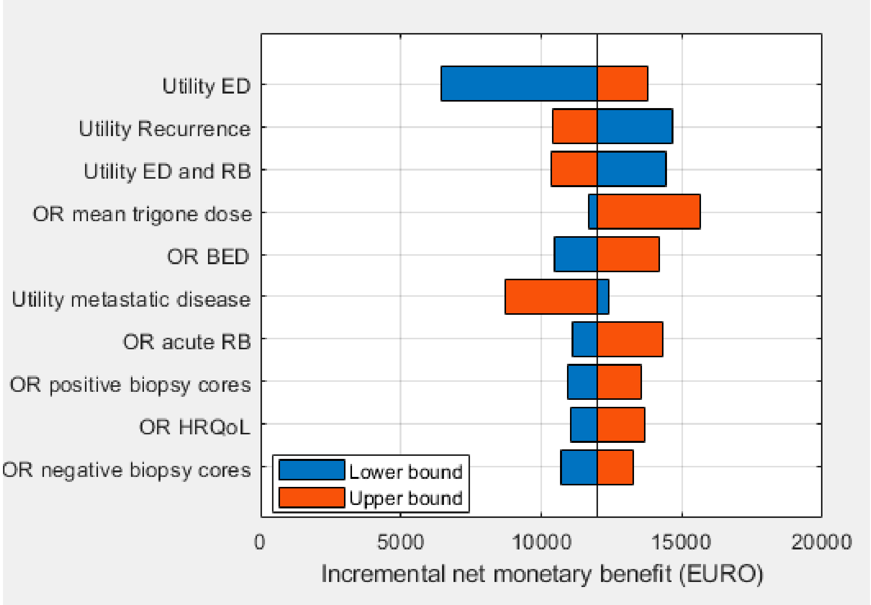

Figure A 1- This figure shows the impact of individual parameters on the incremental net monetary benefit of a decision support system versus randomized treatment. The vertical line represents the deterministic run. ED = erectile dysfunction; OR = odds ratio; BED = biologically effective dose; RB = rectal bleeding; HRQoL = health related quality of life

To test the robustness of the model results, we varied some key assumptions in the model, of which the results are shown in Table A 1. Over the different scenarios the DSS kept a high probability of being cost-effective.

Table A 1- The results of the different scenario analyses

Scenario	*Value*	*TCP diff (%) [95% CI]*	*Cost diff (€) [95% CI]*	*QALY diff (years) [95% CI]*	*P CE (%)*
Discount rate	0%	1.5 [-2.9-5.9]	-478 [-657--324]	0.15 [-0.01-0.29]	99.2
	5%	1.5 [-2.9-5.9]	-296 [-396--196]	0.09 [0.01-0.18]	99.8
	10%	1.5 [-2.9-5.9]	-200 [-265--135]	0.07 [0.00-0.13]	98.6
Dose plan[1]	2.5x28	1.8 [-3.6 -7.3]	-614 [-1376--148]	0.09 [-0.22-0.41]	79.4
	2x35	2.0 [-2.2-6.2]	-147[-832-538]	0.19 [-0.19-0.56]	88.0
	2x37	1.5 [-2.9-5.9]	-308 [-425--192]	0.13 [0.01-0.25]	99.4
Time horizon	5	1.5 [-2.9-5.9]	-78 [-106-50]	0.03 [-0.01-0.07]	97.2
	10	1.5 [-2.9-5.9]	-173[-229--117]	0.07 [-0.00-0.15]	99.2
	15	1.5 [-2.9-5.9]	-260 [-347--172]	0.09 [0.00-0.18]	99.0

	50	1.5 [-2.9-5.9]	-418 [-561--274]	0.17 [-0.00-0.35]	98.6
Costs DSS	20	1.5 [-2.9-5.9]	-304 [-414--194]	0.12 [0.01-0.23]	99.6
	100	1.5 [-2.9-5.9]	-224 [-334--114]	0.17 [-0.0-0.33]	98.6
	250	1.5 [-2.9-5.9]	-74 [-184-26]	0.17 [-0.0-0.33]	99.4
	500	1.5 [-2.9-5.9]	176 [66-286]	0.17 [-0.0-0.33]	99.4

CI = confidence interval; P = probability; EBRT = external beam radiotherapy; DSS = decision support system

[1] Fraction dose x the number of fractions

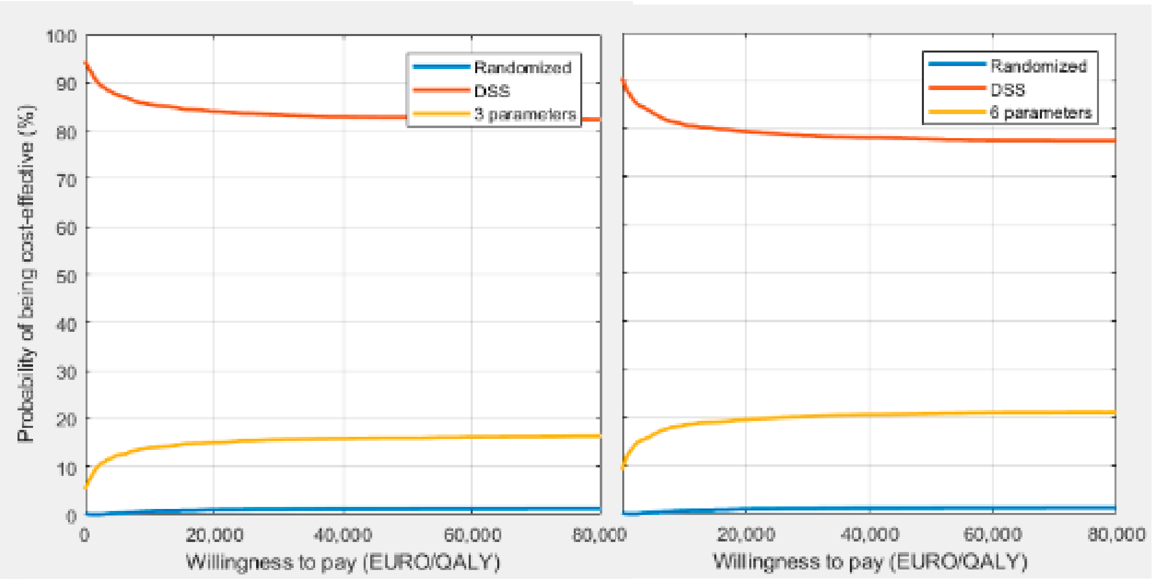

Figure A 2- the cost-effectiveness acceptability curve (CEAC) comparing the randomized treatment allotment strategy to the full decision support system (DSS), and a simplified DSS that utilized only age, prostate specific antigen (PSA) and T-stage.

To test the usefulness of our advanced DSS, we tested a simplified DSS using only three clinical parameters: age, t-stage, and PSA. The simplified DSS had a large probability (85.1%) of being cost-effective compared to the randomized treatment allotment strategy. However, compared to the original DSS, the simplified DSS only had a 16.3% chance of being cost-effective. We expanded the simplified DSS with three extra parameters, including primary Gleason score, diabetes and prior abdominal surgery, and found that this simplified DSS had a 89.1% probability of being cost-effective compared to the randomized strategy, so 4% better than the 3 parameter DSS. However, this 6 parameter DSS still only outperforms the full DSS in 21.1% of the simulations (Figure A 2).
